# Perforated Colon Cancer Associated With Post-operative Recurrent Bowel Perforations

**DOI:** 10.7759/cureus.17655

**Published:** 2021-09-01

**Authors:** Huzaifa Wasanwala, Vladimir Neychev

**Affiliations:** 1 General Surgery, University of Central Florida College of Medicine, Orlando, USA

**Keywords:** colo-rectal cancer, gastro-intestinal perforation, diverticulitis mimic, post-op complications, left hemicolectomy

## Abstract

Colon perforation is a major life-threatening condition associated with high morbidity and mortality, which often develops secondary to complicated diverticulitis and, less commonly, colon cancer. We describe the case of a 51-year-old female who had perforated colon cancer with concurrent diverticulosis. Based on history, physical exam, laboratory, and computed tomography (CT) findings on initial presentation, the patient was diagnosed with acute complicated diverticulitis. Despite medical treatment, the patient’s condition worsened, warranting exploratory laparotomy and a left hemicolectomy with transverse end colostomy creation. Surgical pathology revealed stage IIIC colon cancer without evidence of diverticulitis. The patient underwent eight cycles of adjuvant chemotherapy with FOLFOX (folinic acid, fluorouracil, and oxaliplatin). Over the next year, the patient experienced recurrent bowel perforations requiring repeated surgeries. Perforations were identified in both the small and large bowel on different occasions. Even though neither presented with a clear etiology, possible ischemic, infectious, erosive, and iatrogenic etiologies were on the differential. Our case exemplifies the mounting complications we should be wary of when performing repeated invasive abdominal operations.

## Introduction

Bowel perforation resulting in peritonitis is considered a major life-threatening condition associated with high morbidity and mortality [1]. There are essentially four mechanisms that can lead to perforation in the intestinal tract. These include ischemia (necrosis), infection (appendicitis, diverticulitis), erosion (Crohn’s disease, colon cancer), and physical disruption (iatrogenic, obstruction). In adults, diverticulitis is the most common cause of large bowel perforation [2]. Advanced colon cancer is a less frequent cause of colon perforation. The incidence of colonic perforation due to colon cancer is seen in only about 1.2%-9% of cases [3]. In patients with unremarkable past medical, family, and screening histories, it can be challenging to differentiate a large bowel perforation due to complicated diverticulitis from colon cancer with concurrent diverticulosis. This, in turn, can delay appropriate management, and the correct diagnosis may not be established until final postoperative pathology. We present a patient with left descending colonic perforation secondary to colon cancer. After an index hemicolectomy, the patient encounters a sequela of post-operative complications composed of both small and large bowel perforations following repeated surgical abdominal interventions.

## Case presentation

**Figure 1:**
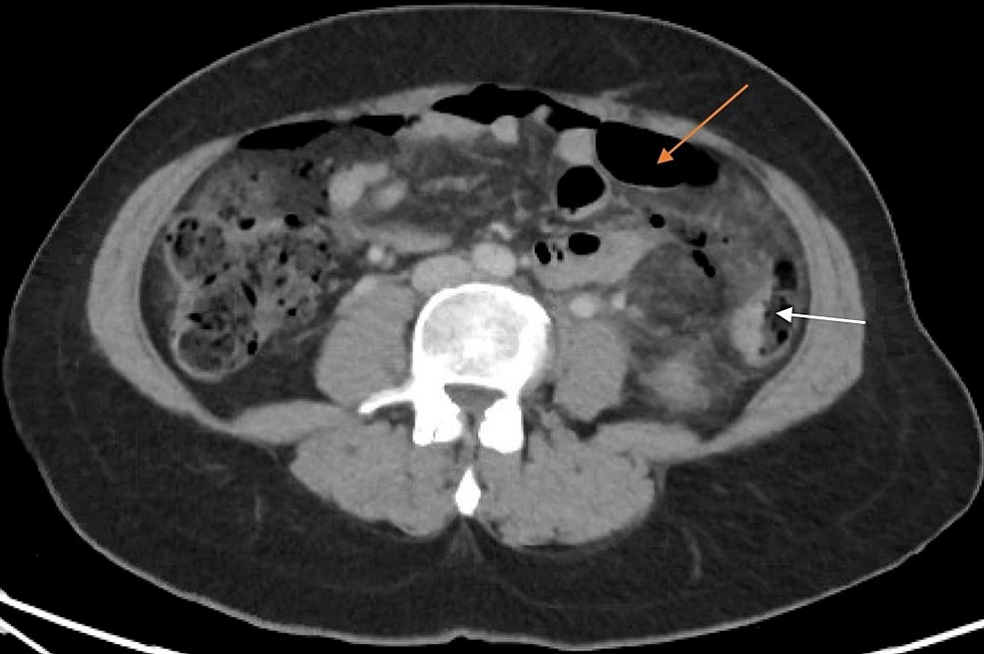
CT abdomen/pelvis without contrast (axial image) showing a moderate amount of pneumoperitoneum (orange arrow) with diverticulosis present (white arrow) in the mid-descending colon

A 51-year-old female with no significant past medical history and surgical history of two cesarean sections presented to the emergency department with a chief complaint of acute onset left lower quadrant (LLQ) abdominal pain. She described it as a constant, cramping pain that radiated to the epigastrium and left upper quadrant (LUQ). She reported nausea, chills, and vomiting. Personal, family, and social history were noncontributory to risk factors for malignancy. On physical exam, her abdomen was tender upon palpation of the LLQ, LUQ, and epigastrium with guarding and rigidity present, along with hypoactive bowel sounds. She was febrile, tachycardic, and hypotensive. Her laboratory results showed a white blood cell (WBC) count of 17.66 K/mm3 and hemoglobin (Hgb) of 10.0 mg/dL. On imaging, computed tomography (CT) abdomen/pelvis scan without contrast showed wall thickening/edema of the mid-descending colon with a perforation at the anterior wall and a moderate amount of pneumoperitoneum (Figure 1). The initial diagnosis after admission workup was acute complicated diverticulitis with a contained micro-perforation consistent with a Hinchey class II diverticulitis. The patient was treated with maximal medical therapy, which consisted of nil per os (NPO), intravenous (IV) fluids, IV antibiotics, and analgesia. She was monitored with serial abdominal CT imaging.

**Figure 2:**
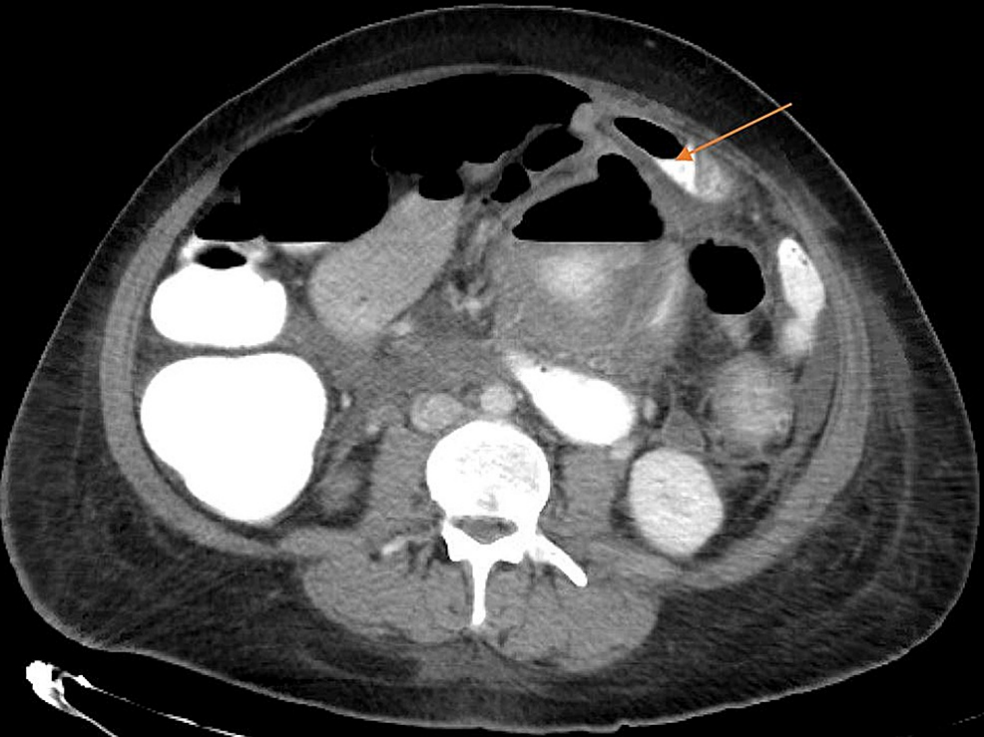
CT abdomen/pelvis with oral contrast (axial cut) showing a large amount pneunoperitoneum and extravasation of contrast in the mid-descending colon (orange arrow)

A week after her initial presentation, the patient experienced severe nausea and emesis followed by rapid worsening of abdominal pain associated with progressive leukocytosis. A stat CT abdomen/pelvis scan revealed an increase of pneumoperitoneum with increased bowel edema and extravasated oral contrast in the left abdomen (Figure 2). It was suspected that the diverticulitis had progressed to Hinchey class IV. An emergency diagnostic laparoscopy was performed and revealed diffuse feculent peritonitis secondary to perforation of the proximal descending colon. Surgery was quickly converted to open, and left hemicolectomy, partial omentectomy, and Hartmann’s pouch were performed. Due to the gross feculent peritoneal contamination, the patient became severely hypotensive and acidotic during the procedure which warranted damage-control abdominal closure with a negative pressure abdominal wound dressing. She was then taken to the intensive care unit (ICU) on ventilation support for appropriate resuscitation.

After two days of resuscitation, a right upper quadrant (RUQ) end transverse colostomy was created. The histopathology report from the resected descending colon specimen at the perforation site revealed a grade two moderately differentiated invasive carcinoma with four out of 17 positive peri-colonic lymph nodes - pT4a pN2a American Joint Committee on Cancer (AJCC) stage IIIC. The initial diagnosis was revised in the context of these pathological findings to perforated colon cancer. Oncology was consulted, and she was started on adjuvant chemotherapy with FOLFOX after temporary subcutaneous port placement.

A year after the index procedure and completion of eight cycles of chemotherapy, the patient underwent pre-operative colonoscopy, with findings of no gross residual malignancy. Subsequently, the colostomy was reversed, tension-free end-to-end colorectal anastomosis was created, and the ostomy site was closed by tertiary intention.

Six days after the colostomy reversal, the patient exhibited worsening leukocytosis, fever, and anemia. Due to excess drainage of peritoneal fluid from the surgical wound and concern for fascial dehiscence, the patient was taken back to the OR for an abdominal washout, partial omentectomy, lysis of small bowel adhesions, and Jackson-Pratt (JP) drain placement. Surgical pathology of the omentum showed necrosis without malignancy. Blood and urine cultures obtained showed no growth.

Two days later, the JP started draining bile, and the patient was taken back for exploratory laparotomy. A small bowel perforation was found in the proximal jejunum and was resected, and a side-to-side functional anastomosis was created. Surgical pathology demonstrated inflammation and edema without malignancy.

**Figure 3:**
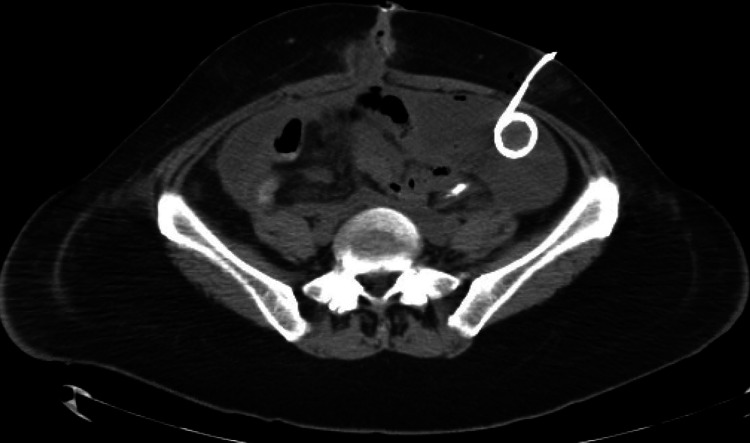
CT abdomen pelvis (axial cut) showing LUQ abdominal abscess with pigtail catheter in place LUQ - left upper quadrant

Five days later, a CT-guided pigtail catheter was placed to drain a LUQ abdominal abscess (Figure 3) after the patient had experienced fever, tachycardia, and rising leukocytosis over the past few days. Cultures grew Candida albicans and Enterococcus faecalis. After a week, the patient recovered well and was discharged with the IR-guided drain in place. Throughout her hospital stay, the patient was receiving total parenteral nutrition (TPN), and her diet was slowly advanced as she began to consume adequate caloric requirements.

**Figure 4:**
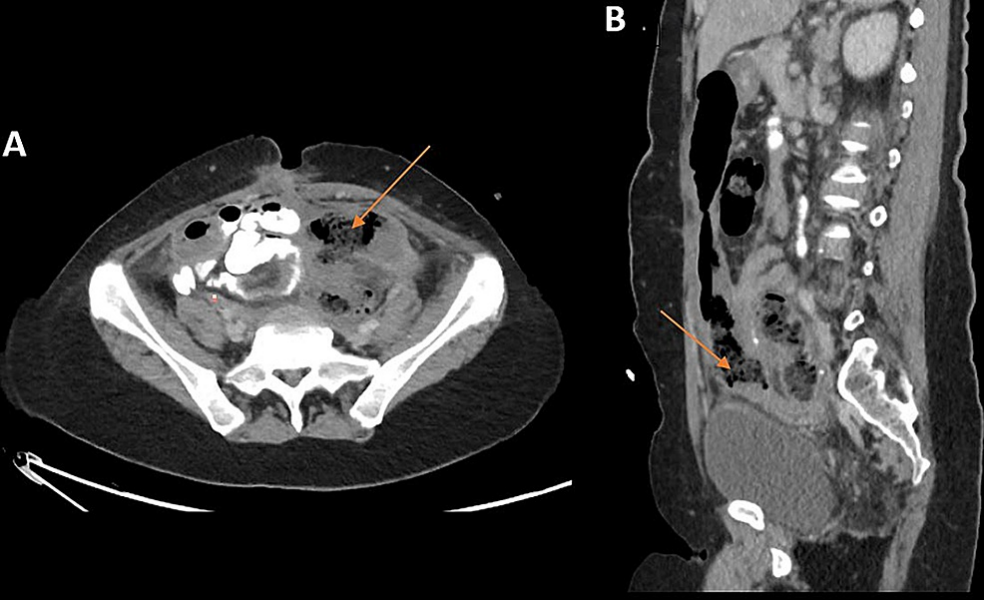
CT abdomen/pelvis with oral contrast (A-axial, B-sagittal) showing an anterior abdominal perforation with pneumoperitoneum, fluid, and feculent material (orange arrows) above the proximal anastomosis

Ten days after discharge, the patient presented to the emergency department with burning epigastric pain, fever, tachycardia, and her drain output changed from minimal serosanguinous output to malodorous fecal material. CT scan of the abdomen/pelvis without contrast showed pneumoperitoneum and fluid collections in the anterior abdomen indicative of perforation (Figure 4). The patient underwent an emergent exploratory laparotomy. Given her multiple recent surgical interventions, a frozen abdomen was encountered. After prolonged lysis of adhesions, an approximately 50% clean, circumferential perforation proximal to the colorectal anastomosis was appreciated. The defect was then repaired primarily, and a protective loop ileostomy for proximal diversion was created. To date, the patient is still recovering from this procedure on the surgical floor.

## Discussion

Differentiating colonic perforation secondary to colon cancer versus complicated diverticulitis is imperative for establishing the correct and timely therapeutic approach and can significantly impact prognosis. In patients who present with colon cancer, those with a free perforation (diffuse peritonitis) have a 19% higher mortality compared to patients with a contained perforation (micro-perforation, abscess) due to increased risk of recurrence and sepsis complications [4,5]. In our case, we presented a patient who had a contained perforation that progressed to a free perforation. Early surgical intervention could have led to more favorable post-operative outcomes with fewer complications. The patient in our case did not provide any significant past medical, family, or social history suspicious of colonic malignancy. She also had never had a screening colonoscopy done before. This made it challenging to delineate between the diagnosis of diverticulitis and colon cancer with concurrent diverticulosis. In 10% of cases, it is difficult to differentiate perforation between these two conditions [6], and in the emergent setting, CT abdominal imaging often becomes the crucial determinant for diagnosis. The more specific CT findings that indicate colon cancer in these cases are the presence of local lymphadenopathy and eccentric thickening of the colon wall [7]. In our patient, the CT scan did not reveal any enlarged mesenteric or retroperitoneal lymph nodes. The patient’s original CT abdominal findings showed bowel wall thickening and edema in the mid-descending colon instead of the sigmoid colon, where diverticulitis most commonly occurs. Although this could have served as an indication to broaden our differential, the patient’s remaining history still favored a diagnosis of diverticulitis.

If the diagnosis of a contained colonic cancer perforation was presumed early on in our case, the therapeutic approach could have been different. Although there is no consensus on how a contained perforation due to colon cancer should be treated [8], the patient may have benefited from an earlier surgical procedure. The patient was hemodynamically stable within the first 24 hours of her initial presentation, making her an appropriate surgical candidate for colon surgery. Early surgical intervention could have prevented diffuse peritonitis and decreased the potential for local tumor recurrence [9].

Following our patient's reverse colostomy, she encountered a series of post-operative perforations. The first one was a perforation in the small bowel on postoperative day eight. Even though the pathology of the resected small bowel showed signs of inflammation and edema, suggesting another potential underlying cause, it is possible that the perforation was created iatrogenically from adhesiolysis following a laparotomy done two days prior. Chronic inflammation could be a driving force for bowel perforation in our patients. Throughout her hospital course, the patient had periods of leukocytosis and fever that could last over a week, indicating an underlying infection. Although blood and urine cultures showed no growth, it is possible that the patient had a localized infection. Also, acute chronic inflammation following repeated laparotomies could be a contributing cause as well. Intestinal manipulation can incite an inflammatory cytokine cascade within the bowel, depressing intestinal motility and locally inhibiting immune function [10]. This can facilitate postoperative infection and weaken bowel wall tissue.

The patient’s second bowel perforation occurred 25 days after the reverse colostomy. The initial suspected cause of her perforation was thought to be an anastomotic leak since the majority of them present within the first 30 days of surgery with an incidence of 1.7% to 14.6% [11]. However, a clean colonic perforation was found proximal to the end-to-end colorectal anastomosis. It is more likely that the perforation here was created iatrogenically from adhesiolysis or thermal injury. Following reverse colostomy, the patient underwent two re-laparotomies and an IR abscess drainage within the span of a month. Her history of multiple invasive abdominal procedures significantly increased her risk for bowel perforation. Extensive adhesion formation from numerous abdominal surgeries alters abdominal anatomy and modifies natural planes [12]. The fusion of multiple intra-abdominal structures makes isolation of portions of the intestines challenging. A single-institution study conducted in the Netherlands showed a ten times higher risk of bowel perforation in patients with a history of three or more prior laparotomies [13]. The delayed presentation of the second perforation (10 days after discharge) suggests an iatrogenic serosal tear during one of her recent abdominal procedures. Her serosal tear could have progressed to a bowel perforation over time from changes in intraluminal pressure and/or shear forces from extensive intra-abdominal adhesions. Other cases in the literature that did report a clean proximal perforation were either due to a stercoral perforation [14] or occurred spontaneously due to chemotherapy agents such as bevacizumab [15]. Our patient did not present with any distal obstruction to the perforation, making stercoral perforation unlikely. Additionally, our patient had not taken bevacizumab as part of her chemotherapy, and her FOLFOX chemotherapy regimen was completed even before the reverse colostomy. FOLFOX therapy itself has not been reported to increase the risk of perforation, making any chemotherapy-related bowel perforation highly improbable.

## Conclusions

In the case of colon perforation, it is important to carefully differentiate between colon cancer and complicated diverticulitis. The treatment approach can vary significantly based on the diagnosis and can greatly impact prognosis. Additionally, recurrent bowel perforations post-operatively after colon surgery can have a wide range of etiologies. In our case, we explored the possible ischemic, infectious, erosive, and iatrogenic etiologies of recurrent bowel perforations that did not present with a clear origin. Our case illustrates the compounding effect that repeated invasive abdominal procedures have on the risk of complications such as bowel perforation.
